# Association between Community Social Capital and Access to Dental Check-Ups among Elementary School Children in Japan

**DOI:** 10.3390/ijerph20010047

**Published:** 2022-12-21

**Authors:** Marie Kobayashi, Yusuke Matsuyama, Nobutoshi Nawa, Aya Isumi, Satomi Doi, Takeo Fujiwara

**Affiliations:** Department of Global Health Promotion, Tokyo Medical and Dental University, Tokyo 163-8001, Japan

**Keywords:** access to dental care, prevention, social capital, child oral health

## Abstract

The association between parental social capital and a child’s access to dental check-ups has been reported, but few studies have focused on dental check-ups. The present study investigated the association between parental social capital and access to dental check-ups among first-grade elementary school children (6–7 years old) in Japan. We analyzed cross-sectional data of first-grade elementary school children (6–7 years old) in Adachi City, Tokyo, Japan. Community social capital (the total score of social trust, cohesion, mutual aid) and child’s dental check-ups (dental check-ups for purposes other than treatment at least once a year) were assessed by questionnaire surveys for parents of the first graders from all 69 elementary schools in 2017 (n = 5260; response rate: 81.6%) and 2019 (n = 5130; response rate: 78.8%). Multilevel Poisson regression analysis, adjusted for children’s age in months, gender, mother’s educational attainment, mother’s employment status, having siblings, living with grandparents, and the density of dental clinics in the school district, was applied. Of the 7936 respondents included in the analysis, 82.7% of children received dental check-ups at least once a year. Individual-level social capital was positively associated with children’s dental check-up utilization (prevalence ratio, PR = 0.935 per one interquartile range, IQR; 95% confidence interval, CI: 0.877, 0.996). Community-level social capital was not significantly associated with children’s dental check-up utilization (PR = 0.934 per one IQR; 95% CI: 0.865, 1.008). Promoting individual-level social capital, but not community-level social capital, may improve dental check-ups among elementary school children in Japan.

## 1. Introduction

Dental caries is the most prevalent disease among children in Japan [1]. It induces various negative consequences in childhood, including pain and discomfort, lower social well-being, poor nutritional health, and difficulty in eating [2]. The association between dental caries and loss of school hours has also been reported [3]. Dental caries experienced in the past strongly predict new cavity development, and dental conditions accumulate throughout life [4]. Thus, optimal and affordable preventive and curative dental care should be provided to every child.

Caregivers contribute to children’s dental care utilization, including treatment and preventive care [5]. In Japan, universal health coverage, including dental care achieved in 1961, and dental services are available with lower copayment than in the USA [6,7]. Dental care costs are further subsidized for children by many local governments [8]. However, dental health insurance in Japan mainly covers treatment rather than prevention, and preventive dental care utilization is low compared to curative treatments [9]. The determinants of preventive dental care include income, parenting style, lack of time, and the working style of caregivers [10].

Social capital is defined as “the resources that are accessed by individuals as a result of their membership of a network or a group” [11]. It is suggested that social capital increases health-related behaviors by enhancing social norms, informal social engagement, and health-related knowledge in the community [12,13,14] and is, thereby, likely to improve healthcare utilization for children [15,16]. Indeed, several studies reported the association between caregivers’ social capital and dental care utilization in children [17,18]. In the US, children whose mothers have lower social capital were less likely to receive dental check-ups in the past year [17]. Another US study reported that caregivers’ financial distress, depressive symptoms, and limited social capital were associated with children’s lower dental care utilization [18]. However, these studies have not separated the two levels of social capital, the individual and community levels, although both are reported to be associated with healthcare utilization in children [15,16]. Therefore, the present study aimed to investigate the association between caregivers’ individual- and community-level social capital and dental check-up utilization in first-grade elementary school children (i.e., 6–7 years old) in Japan.

## 2. Materials and Methods

### 2.1. Study Participants

A cross-sectional study was conducted by pooling two waves of repeated cross-sectional data from the Adachi Child Health Impact of Living Difficulty (A-CHILD) study [19]. In 2017 and 2019, questionnaires were distributed to the caregivers of first-grade children in all 69 public elementary schools in Adachi City, Tokyo, Japan. A total of 5160 and 5130 questionnaires were distributed, and 4428 (response rate: 81.6%) and 4042 (response rate: 78.8%) responded in 2017 and 2019, respectively. These questionnaires were mostly filled by mothers (91%) or fathers (8.2%). After excluding those with missing information on questions on age (n = 144), gender (n = 3), social capital (n = 157), and dental check-ups utilization (n = 314), the data of 7936 respondents (mean age 7.04 years, boys 50.5%) were included in the analysis. Informed consent was obtained from caregivers. This study was approved by the Ethics committee at Tokyo Medical and Dental University (M2016-284).

### 2.2. Measures

The children’s dental check-ups utilization was measured with the following question: “How often does your child have dental checkups in a dental clinic, excluding for treatment purposes?” with response options of “more than 3 times per year”, “once or twice per year,” and “none.” We dichotomized these categories into “once or more per year (coded 0)” and “none (coded 1)” to indicate a lack of dental check-up use.

Individual-level social capital, i.e., social trust, cohesion, and mutual aid, were measured by using the following three questions: “Do you agree or disagree with the following statements? (1) People in your community can be trusted; (2) this community is close-knit; (3) people in your community are willing to help their neighbors,” with a 5-point Likert scale of 1, strongly agree; 2, somewhat agree; 3, neither agree nor disagree; 4, somewhat disagree, and 5, strongly disagree. We followed the approach of a previous study [20]; total scores of these 3 questions were reversed so that a higher score indicates higher individual-level social capital. The average score of individual-level social capital by year and school district was used as community-level social capital, centered at the school-level mean. In the regression analysis, individual- and community-level social capital scores were divided by the interquartile range (IQR) for interpretation.

Potential confounders were also measured by questionnaire: child’s age and gender, mother’s educational attainment (high school or less, some college or more, other/unknown), mother’s employment status (full-time working, part-time/self-employment/side job/other, not working), having siblings (yes, no), and living with grandparents (yes, no). We focused on maternal characteristics based on previous studies that also used the same Adachi Child Health Impact of Living Difficulty (A-CHILD) study data [21,22,23]. Also, as the density of dental clinics is associated with dental care utilization [24,25], the number of dental clinics per 1000 population in each school district was adjusted [26].

### 2.3. Statistical Analysis

Two-level multilevel Poisson regression analysis with random intercepts (level 1: children, level 2: school district) was performed to investigate the association between individual- and community-level social capital and a child’s dental check-up utilization. The level for the survey year was not considered because the clustering within the survey year was too weak to be estimated. Three models were constructed by sequentially adding independent variables: individual- and community-level social capital (model 1); adjusting for child age and gender, mother’s educational attainment, mother’s employment status, having siblings, living with grandparents (model 2); and further adjusting for the density of dental clinics (model 3). Missing values on covariates were coded as dummy variables and included in the analysis. All analyses were performed using Stata SE version 15 (StataCorp. 2017. College Station, TX, USA: StataCorp LLC).

## 3. Results

Table 1 describes the demographic characteristics of the participants. Among them, 17.3% did not receive dental check-ups in the past year. The individual- and community-level social capital scores were significantly lower in children without dental care for prevention than in those with dental care for prevention. The prevalence of children without dental care for prevention was higher in the 2019 survey, with boys and mothers with lower educational attainment.

Table 2 reports the association between individual- and community-level social capital and children’s dental check-up utilization. Model 1 showed that high individual-level social capital was associated with receiving dental check-ups (PR: 0.925, 95% CI: 0.868–0.985). The community-level social capital was not significantly associated with children’s dental care for prevention (PR: 0.926, 95% CI: 0.855–1.002). Similar results were observed after adjusting for individual-level covariates (Model 2; PR for individual-level social capital: 0.935, 95% CI: 0.877–0.996; PR for community-level social capital: 0.938, 95% CI: 0.868–1.013). The results remained similar after adjusting for the density of the dental clinic (Model 3; PR for individual-level social capital: 0.935, 95% CI: 0.877–0.996; PR for community-level social capital: 0.934, 95% CI: 0.865–1.008).

## 4. Discussion

The present study examined the association of individual- and community-level social capital with dental check-ups among first-grade elementary school children (6–7 years old) in Japan. Individual-level social capital was associated with children’s dental care use for prevention, but community-level social capital was not. This result means that if the social capital score increases, people tend to have more chances to receive dental check-ups.

Some studies have reported the association between individual-level social capital and preventive dental care utilization of children [17,18,27]. In the US, caregivers’ low social capital has been reported as a barrier for children visiting dentists [18]. They used similar measures of social capital to ours, such as neighborhood trust and mutual aid, but did not separate preventive dental care and dental treatment. Another study from the US found a positive association between mothers’ perceived social capital and dental care for prevention among children aged 0–17 years old [17]. The present study investigated community-level social capital as a determinant of dental care utilization of children. However, Santoso et al. (2020) reported a positive association between structural, but not cognitive, social capital in the community and dental care use among people aged 15 years or older [27]. The present study revealed that caregivers’ perceived social capital at the individual level, but not the community level, was associated with dental check-up use by young children.

Social capital is supposed to enhance the spreading of health information and healthy norms in the community, leading to better health and health-related behaviors [28]. Therefore, in our study, caregivers with high social capital may easily obtain information about child dental care. Under the healthcare system in Japan, people have the option to choose their dentist. A previous study reported that 5.8% of caregivers have difficulty deciding to which dental clinic to take their children [10]. Exchanging information and sharing their children’s experiences at dental clinics may help caregivers facilitate decision-making. However, to our knowledge, no studies have directly examined the pathway by which high social capital led to better dental care utilization for prevention or treatment. A future longitudinal study is needed to reveal the mechanism.

There are some limitations in this study. First, we defined preventive dental care as having dental checkups in dental clinics, excluding treatment purposes. Thus, it is unknown what procedure is included; for example, it may or may not contain topical fluoride utilization or dental fissure sealant. It could also be difficult for some caregivers to distinguish whether the purpose of their children’s dental visit was for treatment or prevention. Second, the school district may not represent the caregiver’s and children’s community because they could choose schools in different districts, although the majority usually choose schools according to the district. Third, as this is a cross-sectional study, the causal relationship remains unknown. Lastly, the participants lived in a city, Tokyo. Thus, different results might be obtained if similar studies were conducted in other areas in Japan, although we have adjusted for the density of dentists. Further research is needed to confirm this finding and see if it is generalizable to other areas.

## 5. Conclusions

In conclusion, our study found a positive association between individual-level and community-level social capital and dental check-ups of first-graders in Japan.

## Figures and Tables

**Table 1:** Demographic characteristics of study participants (N = 7936).

		Having Dental Check-Ups in the Past Year		
	Total	Yes	No	
	N = 7936	N = 6561 (82.7%)	N = 1375 (17.3%)	
	N (%) or Mean (SD)	N (%) or Mean (SD)	N (%) or Mean (SD)	*p*-Value
Survey year				0.043
2017	3891 (49.0%)	3251 (83.6%)	640 (16.4%)	
2019	4045 (51.0%)	3310 (81.8%)	735 (18.2%)	
Age (month)	84.47 (3.47)	84.55 (3.46)	84.12 (3.50)	<0.001
Gender				0.048
Boy	4009 (50.5%)	3281 (81.8%)	728 (18.2%)	
Girl	3927 (49.5%)	3280 (83.5%)	647 (16.5%)	
Mother’s educational attainment				<0.001
High school or less	2485 (31.3%)	1970 (79.3%)	515 (20.7%)	
Some college or more	5205 (65.6%)	4396 (84.5%)	809 (15.5%)	
Other/Unknown	72 (0.9%)	57 (79.2%)	15 (20.8%)	
Missing	174 (2.2%)	138 (79.3%)	36 (20.7%)	
Mother’s employment status				0.096
Full-time	1851 (23.3%)	1526 (82.4%)	325 (17.6%)	
Part-time/self-employment/side job/other	3718 (46.8%)	3093 (83.2%)	625 (16.8%)	
Not working	2254 (28.4%)	1858 (82.4%)	396 (17.6%)	
Missing	113 (1.4%)	84 (74.3%)	29 (25.7%)	
Only child				0.570
No	6324 (79.7%)	5236 (82.8%)	1088 (17.2%)	
Yes	1612 (20.3%)	1325 (82.2%)	287 (17.8%)	
Living with grandparents				0.780
No	7164 (90.3%)	5920 (82.6%)	1244 (17.4%)	
Yes	772 (9.7%)	641 (83.0%)	131 (17.0%)	
Individual-level social capital	10.18 (2.29)	10.22 (2.27)	10.01 (2.42)	0.002
Community-level social capital	10.17 (0.45)	10.18 (0.45)	10.15 (0.44)	0.018
Dental clinic per 1000 population	0.56 (0.36)	0.56 (0.36)	0.54 (0.33)	0.028

Abbreviations: standard deviation, SD.

**Table 2:** Association between social capital and access to dental check-ups of children; multilevel Poisson regression analysis; level 1: individual (N = 7936); level 2: school (N = 69).

	Model 1	Model 2	Model 3
	PR (95% CI)	PR (95% CI)	PR (95% CI)
**Fixed effects**			
Individual-level social capital (per IQR)	0.925 (0.868, 0.985)	0.935 (0.877, 0.996)	0.935 (0.877, 0.996)
Community-level social capital (per IQR)	0.926 (0.855, 1.002)	0.938 (0.868, 1.013)	0.934 (0.865, 1.008)
Age (month)		0.973 (0.958, 0.988)	0.973 (0.958, 0.988)
Gender			
Boy		ref.	ref.
Girl		0.911 (0.819, 1.013)	0.911 (0.819, 1.013)
Mother’s educational attainment			
High school or less		ref.	ref.
Some college or more		0.769 (0.686, 0.862)	0.773 (0.690, 0.868)
Other/Unknown		1.010 (0.604, 1.691)	1.015 (0.606, 1.698)
Missing		0.908 (0.627, 1.314)	0.911 (0.630, 1.319)
Mother’s employment status			
Full-time		ref.	ref.
Part-time/self-employment/side job/other		0.890 (0.776, 1.020)	0.888 (0.775, 1.018)
Not working		0.937 (0.808, 1.088)	0.936 (0.806, 1.086)
Missing		1.320 (0.869, 2.007)	1.318 (0.867, 2.003)
Only child			
No		ref.	ref.
Yes		1.016 (0.891, 1.158)	1.019 (0.894, 1.162)
Living with grandparents			
No		ref.	ref.
Yes		0.925 (0.770, 1.110)	0.925 (0.770, 1.110)
Dental clinic per 1000 population			0.855 (0.699, 1.046)
**Random effects on intercept**			
Community-level variance (95% CI)	0.041 (0.010, 0.072)	0.035 (0.006, 0.063)	0.032 (0.005, 0.060)

Abbreviations: prevalence ratio, PR; confidential interval, CI; reference, ref; interquartile range, IQR.

## Data Availability

The data that support the findings of this study are available on request from the corresponding author. The data are not publicly available due to privacy or ethical restrictions.
